# Mild hypercholesterolemia impacts achilles sub-tendon mechanical properties in young rats

**DOI:** 10.1186/s12891-023-06375-0

**Published:** 2023-04-12

**Authors:** Charlie M. Waugh, Rouhollah Mousavizadeh, Jenny Lee, Hazel R. C. Screen, Alexander Scott

**Affiliations:** 1 Dept. Physical Therapy, Faculty of Medicine, University of British Columbia, Vancouver BC, Canada; 2 School of Engineering and Materials Science, Queen Mary, University of London, London, U.K.

**Keywords:** Biomechanical properties, Lipids, Histology, Force relaxation, Xanthoma

## Abstract

**Background:**

Hypercholesterolemia is associated with tendon pathology, but the reasons underpinning this relationship are not well understood. Cholesterol can accumulate in the tendon non-collagenous matrix which may affect both global and local tissue mechanics. Changes to the local strain environment within tendon may have significant implications for mechanosensitive tenocytes. Here, we investigated the association between elevated blood cholesterol and presence of tendon lipids in the Achilles tendon. We expected lipids to be localised in the proteoglycan-rich inter-sub-tendon matrix (ISTM), therefore we also sought to examine the impact of this on the biomechanical and viscoelastic properties of the ISTM.

**Methods:**

The Achilles tendons of 32 young wild-type (SD) and 32 apolipoprotein E knock-out rats (*ApoE*^*−/−*^) were harvested at 15.6 ± 2.3 weeks of age. 32 specimens underwent histological examination to assess the distribution of lipids throughout sub-tendons and ISTM. The remaining specimens were prepared for biomechanical testing, where the ISTM between the gastrocnemius and soleus sub-tendons was subjected to shear load mechanical testing. A sub-set of tests were video recorded to enable a strain analysis.

**Results:**

*ApoE*^*−/−*^ serum cholesterol was double that of SD rats (mean 2.25 vs. 1.10 mg/ml, p < 0.001) indicating a relatively mild hypercholesterolemia phenotype. Nonetheless, we found histological evidence of esterified lipids in the ISTM and unesterified lipids in the sub-tendons, although the location or intensity of staining was not appreciably different between rat strains. Despite a lack of observable histological differences in lipid content between groups, there were significant differences in the mechanical and viscoelastic behaviour of the Achilles sub-tendon matrix.

**Conclusion:**

Even slightly elevated cholesterol may result in subtle changes to tendon biomechanical properties and hence injury risk. The young age of our cohort and the mild phenotype of our *ApoE*^*−/−*^ rats are likely to have limited our findings and so we also conclude that the *ApoE*^*−/−*^ rat model is not well suited for investigating the biomechanical impact of tendon xanthomas on Achilles sub-tendon function.

**Supplementary Information:**

The online version contains supplementary material available at 10.1186/s12891-023-06375-0.

## Introduction

Individuals with high cholesterol demonstrate elevated levels of low-density lipoproteins (LDL), which are directly associated with the likelihood of developing atherosclerotic plaques; infiltration and retention of LDL in vessel walls promotes fatty plaque development. Hypercholesterolemia is also associated with tendon pathology and increased injury prevalence [1, 2]. The pathophysiological mechanisms underpinning the formation of tendon xanthomas (cholesterol deposits in tendons) are not well characterised [3] but are hypothesised to be similar to atherosclerosis [4].

Tendons are fibrous, rope-like tissues that transfer muscle forces to the skeleton, often withstanding large and non-uniform forces [5–8]. The Achilles tendon is the largest and strongest tendon in the body, composed of three sub-tendons, arising from the separate associated muscle bellies of the triceps surae. Sliding between these sub-tendons is critical for healthy Achilles function, enabling non-uniform tendon extension in response to loading [9]. Indeed, a loss of sub-tendon sliding has been shown to occur with ageing and has been associated with tendon pathology [10, 11].

The inter sub-tendon matrix (ISTM) is a specialized non-collagenous matrix (NCM) interspersed between sub-tendons of the Achilles tendon [12]. It may be particularly receptive to LDL accumulation due to its blood supply [13] and high glycosaminoglycan (GAG) content [14–16]. Changes to ISTM composition and structure are likely to affect ISTM, and subsequently tendon, mechanical behaviour [11, 17]. For example, GAG-depleted tendons demonstrate reduced inter-fibrillar sliding [18, 19] and increased viscoelasticity [20], which may have implications for the tendon’s fatigue properties and ability to respond to repetitive loading [21].

Energy storing tendons, such as the Achilles and quadriceps tendons, experience greater loads more frequently and have a lower safety factor than positional tendons [22]. They also more commonly present with xanthomas [23, 24]. For example, 40% of ruptured and 20% of control quadriceps tendons demonstrate histological evidence of intratendinous lipids [25, 26]. This lipid accumulation is possibly a result of having a greater non-collagenous protein content [22, 27, 28] and has the potential to alter the local mechanical environment and therefore tenocyte mechanosensing. It is likely, therefore, that intratendinous lipid accumulation would negatively impact tissue maintenance.

An association between rotator cuff tears and high cholesterol has been previously reported [29], and recently co-location of cholesterol deposits and collagen fibrils was found to cause physical fibril damage and subsequent tendon weakening via an unidentified mechanism [30]. Given the prevalence of tendon xanthomas with familial hypercholesterolemia [31, 32], it is of practical importance to investigate the biomechanical impact of lipid accumulation on tendon mechanics.

First, we hypothesized that elevated blood cholesterol would be associated with an increased presence of tendon lipids, localised to the vascularized and proteoglycan-rich inter sub-tendon matrix (ISTM). Second, we hypothesised that the presence of lipid in the ISTM would impact ISTM mechanical properties and thus local tissue strains within tendon.

We used an established rat knock-out model to mimic familial hypercholesterolemia (SD- *ApoE*^*tm1sage*^, Horizon Discovery, Saint Louis, MO, USA) to examine our hypotheses. This model brings about mild-moderate hypercholesterolemia without feeding a high-fat diet [33] through a defect in Apolipoprotein E (ApoE) - a critical protein used for transporting lipoproteins, fat-soluble vitamins, and cholesterol around the body. This model avoids any comorbidities associated with feeding a high fat diet that might otherwise affect our findings.

## Methods

### Experimental animals

Ethical approval information is detailed at the end of the manuscript. We used *ApoE*^*−/−*^ rats (SD- *ApoE*^*tm1sage*^, Horizon Discovery, Saint Louis, MO, USA) to examine the effect of elevated blood cholesterol on tendon lipid content; Sprague Dawley wild-type rats (SD; Charles River Laboratories, Wilmington, MA, USA) were used as control animals. Breeding pairs were co-housed when animals were 24 and 14 weeks, respectively. Rat husbandry was carried out by certified animal laboratory technicians. Rats were maintained on a 24-hr light/dark cycle between 21 and 24 °C. All rats were fed regular chow (5% fat; PicoLab Rodent Diet 20: 5053, LabDiet; Richmond, IN, USA) for the study duration. Cages contained nesting material and environmental enrichment (plastic tubes, chew toys). Litters were weaned and sexed at 21 days old and sorted into group housing (2–3 per cage) located in a room separate from the breeders [34].

A total of 60 SD rats and 50 *ApoE*^*−/−*^ rats were bred from the breeding program; 32 SD rats and 32 *ApoE*^*−/−*^ rats were used in this study (all animals were used in related experiments [34]). No inclusion or exclusion criteria were established, and we attempted to use equal male/female ratios throughout. All rats underwent a patellar tendon injury surgery at approximately 12 weeks of age [34]. During surgery, animals’ ears were notched for identification. This was done in no particular order; thus experimenters were blinded from tissue identification until all experiments were complete. On recovering from anaesthesia, animals resumed full mobility and were allowed to continue normal cage activity until they were sacrificed.

### Tissue harvesting

Rats were euthanized between 13 and 18 weeks of age. Animals underwent anesthesia with a mixture of isoflurane (2–3%) and oxygen (100%) delivered via nose cone. 0.5ml of blood was collected in a lithium heparin tube via cardiac puncture just prior to euthanization with carbon dioxide, with death assured by cervical dislocation. Achilles tendons were harvested immediately thereafter.

### Blood lipids

Total blood serum cholesterol (free cholesterol and cholesteryl esters) was assessed with a fluorometric cholesterol assay (Invitrogen, cat. no. A12216).

### Histology

Histological sections were obtained from 32 samples (16 SD, 16 *ApoE*^*−/−*^) to allow a reasonable evaluation of lipid content in each group. The Achilles tendon was dissected away from the triceps surae muscle group with a sterile blade, just proximal to the gastrocnemius medialis muscle-tendon junction and at the calcaneus, then embedded in OCT in cryomolds with the orientation noted. Samples were flash frozen by placing on a liquid nitrogen-cooled metal block and kept at -80 °C until required. Frozen tissue was sectioned on a cryostat at -22 °C at 10 μm thicknesses in the sagittal plane and recovered onto gelatin-alum coated slides; 3–5 sections from each sample were recovered onto each slide. Sections were then fixed for 2 min in 95% EtOH and washed in dH_2_O.

Oil Red-O (ORO) staining was used to visualise esterified lipid content in the tissue section [35], and cell nuclei counterstained with Gill’s Haematoxylin. Sections were imaged using a Leica light microscope (ZEISS Axiophot and AxioCam ICc1, Carl Zeiss AG, Oberkochen, Germany). The Achilles fat pad provided a positive ORO staining control. Filipin (F334451, LKT Laboratories, Inc., St Paul, MN, USA) was used to visualise unesterified lipids in the tissue [36]. Filipin III powder was dissolved in DMSO (5 mg/mL) and the stock solution diluted 1:100 with PBS for a working solution. Sections were incubated with the fluorescent stain for 30 min, washed twice in PBS and counterstained with Propidium Iodide containing mounting media (ab104129, AbCam, Cambridge, UK) prior to imaging (ZEISS Observer A1 and AxioCam ICm1, Carl Zeiss AG, Oberkochen, Germany). Images were digitally captured, and post-processing image adjustments were made within the camera’s proprietary software (Zen 3.4, Carl Zeiss Microscopy GmbH). ISTM width was estimated in ImageJ [37] from calibrated images of at least two histological sections per sample, and the median measurement taken to represent width.

### Biomechanics

**Fig. 1:**
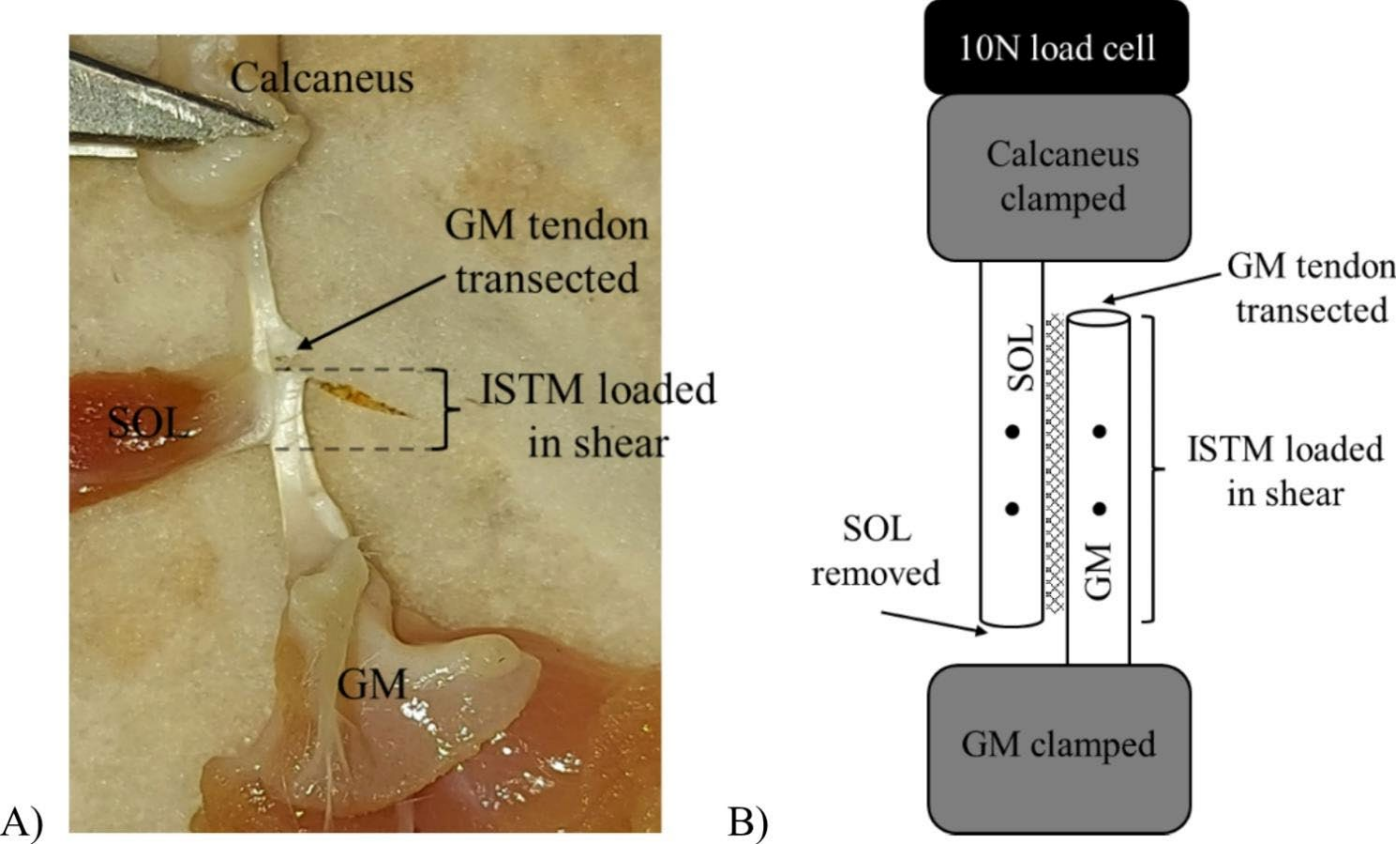
A) Sample preparation and B) sample configuration when mounted in testing rig. Tensile forces from loading protocols loaded ISTM (hashed area) in shear. Strain analysis was completed using (black) marks on sub-tendons

32 rats (16 SD, 16 *ApoE*^*−/−*^) provided 64 Achilles tendons for investigating ISTM mechanical properties; this sample size was based on previous work and takes into account a reasonable likelihood of dissection and sub-tendon isolation errors [9]. The Achilles tendon was removed by dissecting proximally at the triceps surae group muscle belly and distally by retaining a portion of the calcaneus. Tendons were wrapped in PBS-soaked gauze and stored in airtight containers at -20 °C until required. Upon thawing at room temperature, the plantaris and gastrocnemius lateralis soft tissues were removed, leaving the gastrocnemius medialis (GM) and soleus (SOL) bound together. The remaining muscles were divided down to their tendon’s junction. Distal separation of the sub-tendons was achieved by identifying and gently piercing the inter sub-tendon matrix (ISTM) with a thin-gauge needle proximal to the calcaneal attachment, with the aid of magnification glasses. The GM tendon was then carefully transected, leaving only the SOL attached to the calcaneus (Fig. 1A). Finally, the SOL muscle was removed, and GM muscle tissue scraped off the aponeurosis with the dull side of a sterile blade, leaving behind a fan of aponeurosis tissue. The calcaneus and the GM aponeurosis were secured in sandpaper-lined clamps to increase friction and minimise tissue slippage, to load the ISTM in shear (Fig. 1B). Samples were then loaded in mechanical testing rig (5960 series, Instron, Norwood, MA *or* ElectroForce 5100, Bose Corporation, Eden Prairie, MN, USA) fitted with a 10 N load cell.

To determine the mechanical behavior of the ISTM, each sample was loaded to 0.1 N to remove slack and identify the starting point of the test. Samples were then immediately loaded for 10 loading-unloading cycles at 0.333 Hz between 0.1 and 0.5 mm displacement using a triangular waveform without preconditioning. Samples were then momentarily returned to 0 mm extension before holding a 0.5 mm extension for 5 min to assess their force relaxation properties. Finally, samples were loaded to failure at an extension rate of 0.1 mm/s. Tests were always administered in this order and at room temperature (range 21–24°, 40–61% humidity). Load and displacement of all tests were sampled at 200 Hz. Samples were frequently sprayed with PBS throughout the loading protocols to maintain hydration. After mechanical testing, the GM and SOL sub-tendons were manually separated to assess whether they had been correctly isolated, and test data only retained if isolation had been correctly implemented.

Sample shear properties were calculated for each loading-unloading cycle and averaged over the last 5 cycles. Stiffness was estimated from the gradient of the force-extension curve using a linear regression model fitted between 50 and 90% of the loading curve (mean R^2^ 0.988 ± 0.024). Hysteresis was calculated as the area contained between the loading and unloading curve. Coefficients of variation (CV) were calculated for stiffness, hysteresis and peak force over the last 5 cycles to confirm cycle consistency (CVs of 0.9, 4.0 and 1.7, respectively). The decrease in peak force at peak extension between cycles 1 and 10 was calculated relative to cycle 1 and hereafter termed cyclic force decrement (%). Similarly, force-relaxation was quantified as the force decay during the 5-min static hold relative to the force at initial extension (%). Maximal stiffness was obtained by calculating continual stiffness over a 5 data-point moving average window of the force-displacement data of the ramp-to-failure test and taking the largest value. Ultimate strength (maximum force) and extension were also determined from the ramp-to-failure tests.

Sub-tendon strains were assessed during the shear test on a subset of samples (8 SD, 8 *ApoE*^*−/−*^) to explore the relative mechanical stretch of the ISTM and sub-tendons. Overall sample extension was determined from the grip-to-grip length between the clamps. Localised extension of GM and SOL sub-tendons were tracked using two permanent ink marks made to the mid-portion of each sub-tendon prior to experimentation (Fig. 1B), with analysis of markers across the two sub-tendons also enabling a local analysis of shearing of the two sub-tendons (hereafter termed shear). A DSLR camera (Panasonic HC-V720) mounted on a tripod and positioned at the same height as the testing sample was used to record marker movements (60 frames/s, frame size 720 × 1280). Videos were calibrated using known in-plane dimensions and manually digitised (Tracker v5.1.5, physlets.org/tracker).

### Data Analysis and Statistics

Once data was processed, ear notch ID was matched to rat ID (strain, sex). Statistical analysis was done using SPSS statistical software (v23, IBM Corp., Armonk, NY, USA). Blood cholesterol levels and weights were compared between rat strains with independent t-tests. ORO and Filipin staining of histological sections were graded semi-quantitatively with a 5-scale rating system (0, 1, 2, 3, or 4), where 0 = none and 4 = intense staining [38]. Grading was completed for fibres (sub-fascicle) and the ISTM. In addition, confidence in grading was also scored, where 0 = none and 4 = high; any sections receiving a 0–1 confidence score were removed from further analysis. Grading was completed blinded to rat strain on two occasions by one grader 6–7 days apart; the averaged scores were compared between rat strains with independent t-tests. Intra-rater reliability was assessed by computing intra-class correlation (ICC) estimates and 95% confident intervals (CI) based on a mean-rating (*k* = 2), absolute-agreement, 2-way mixed-effects model [39]. Achilles ISTM biomechanical properties were examined for normality using Shapiro-Wilks tests; not-normally distributed dependent variables were log-transformed to meet the null hypothesis for normality and differences between groups were then examined with one-way ANOVAs and follow-up t-tests.

## Results

### Cholesterol/weight/macroscopic diffs

64 animals (32 SD, 32 *ApoE*^*−/−*^) were used in this study. Mean ± s.d. animal age at euthanasia was 15.6 ± 2.6 weeks. SD males were marginally heavier than *ApoE*^*−/−*^ males at injury date (mean ± s.d. 388.2 ± 37.9 g vs. 357.8 ± 38.1 g, *p* = 0.02) but weights of SD and *ApoE*^*−/−*^ female rats did not significantly differ (mean ± s.d. 259.4 ± 16.1 g vs. 248.7 ± 28.2 g, *p* = 1.0). At the point of euthanasia, *ApoE*^*−/−*^ total cholesterol (TC) was over double that of SD rats (mean ± s.d. 2.25 ± 0.30 vs. 1.10 ± 0.54 mg/ml, p < 0.001). There were no sex differences in TC within each strain. There were no obvious differences among groups regarding the macroscopic appearance of the tissue.

### Histology

**Fig. 2:**
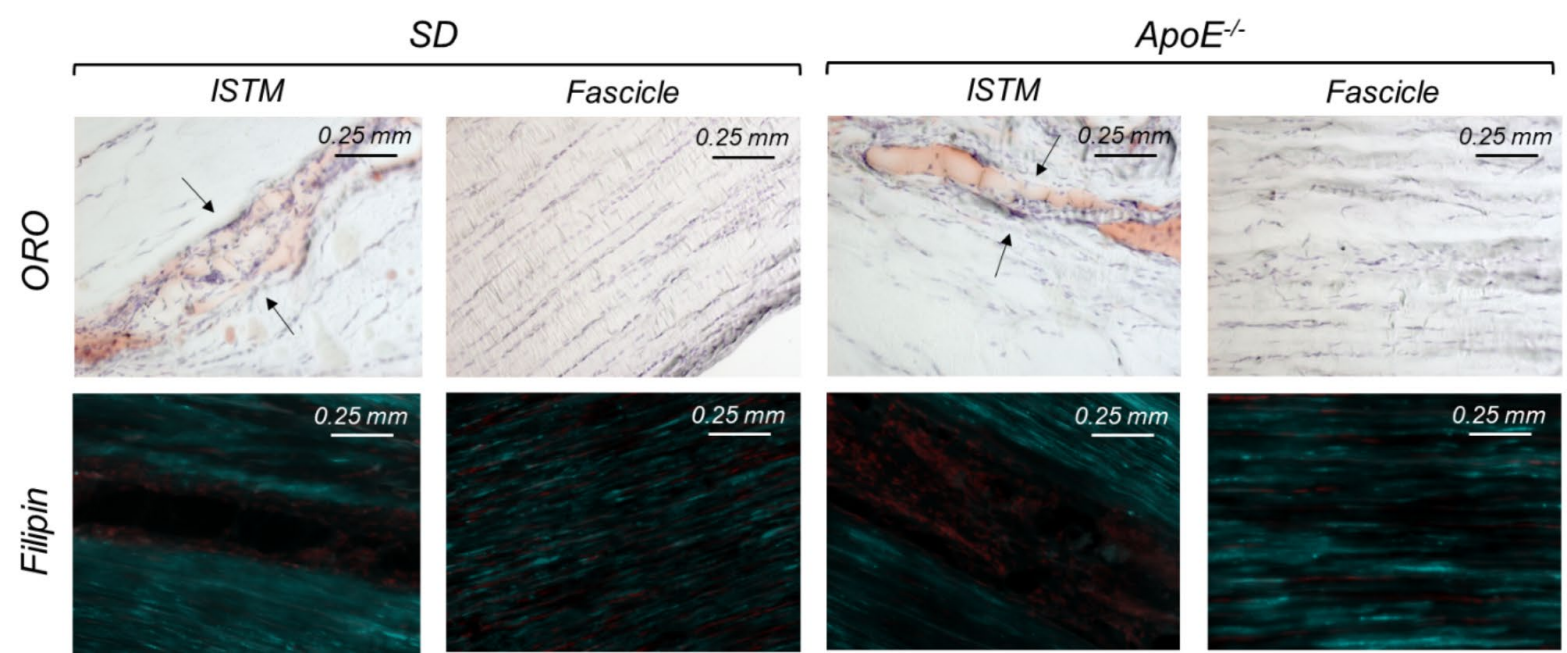
Lipid staining in the ISTM and sub-fascicle. Positive ORO staining was frequently identified in the ISTM of both SD and ApoE^−/−^ rats but rarely between fibres. The opposite was true for filipin staining (cyan; nuclei counterstained red); positive staining was diffuse amongst fibres but not typically observed in the ISTM

**Fig. 3:**
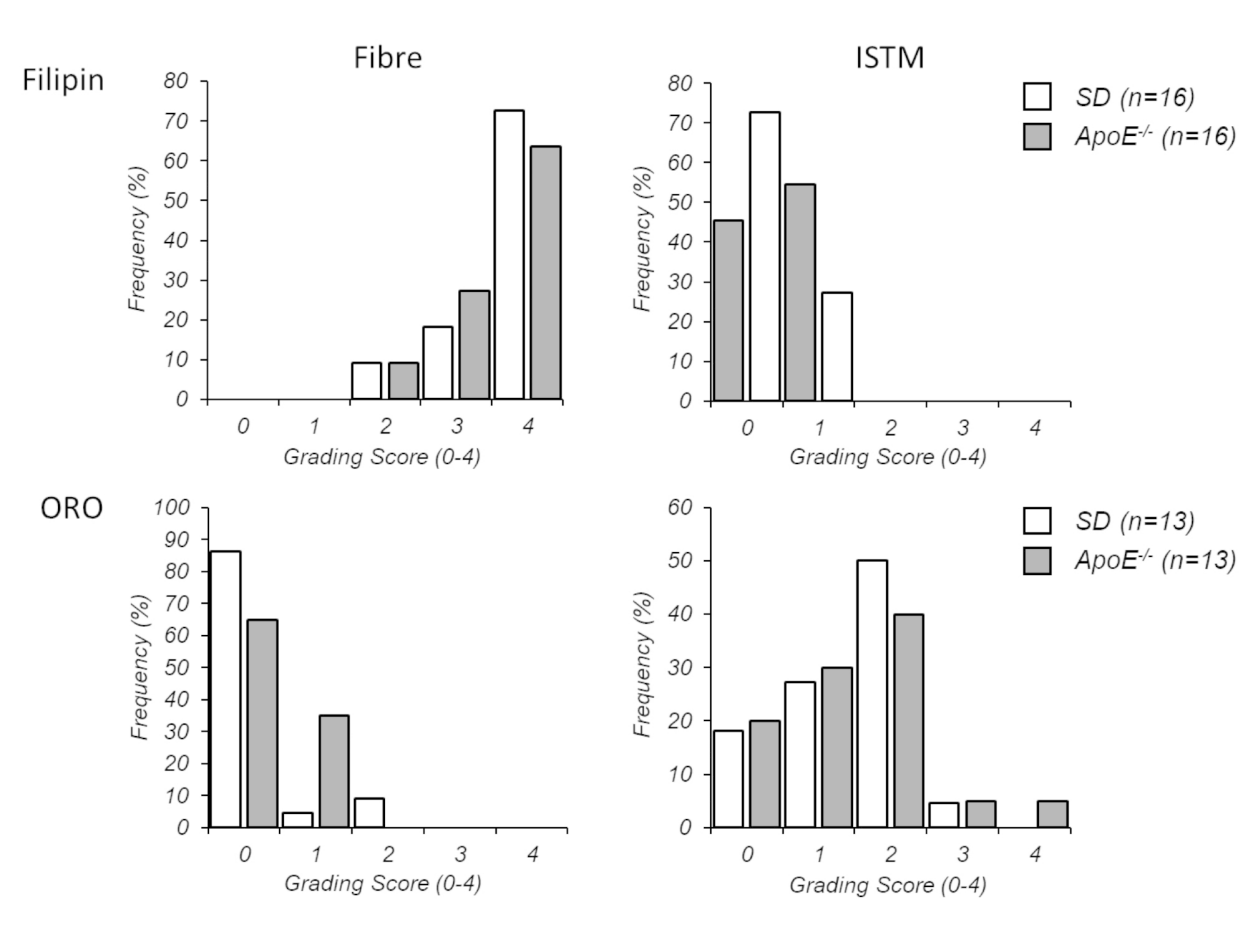
Frequency histograms demonstrating spread of Oil Red-O (ORO) and Filipin staining scores (0 = none, 1 = minimal, 2 = mild, 3 = moderate, 4 = high)

**Fig. 4:**
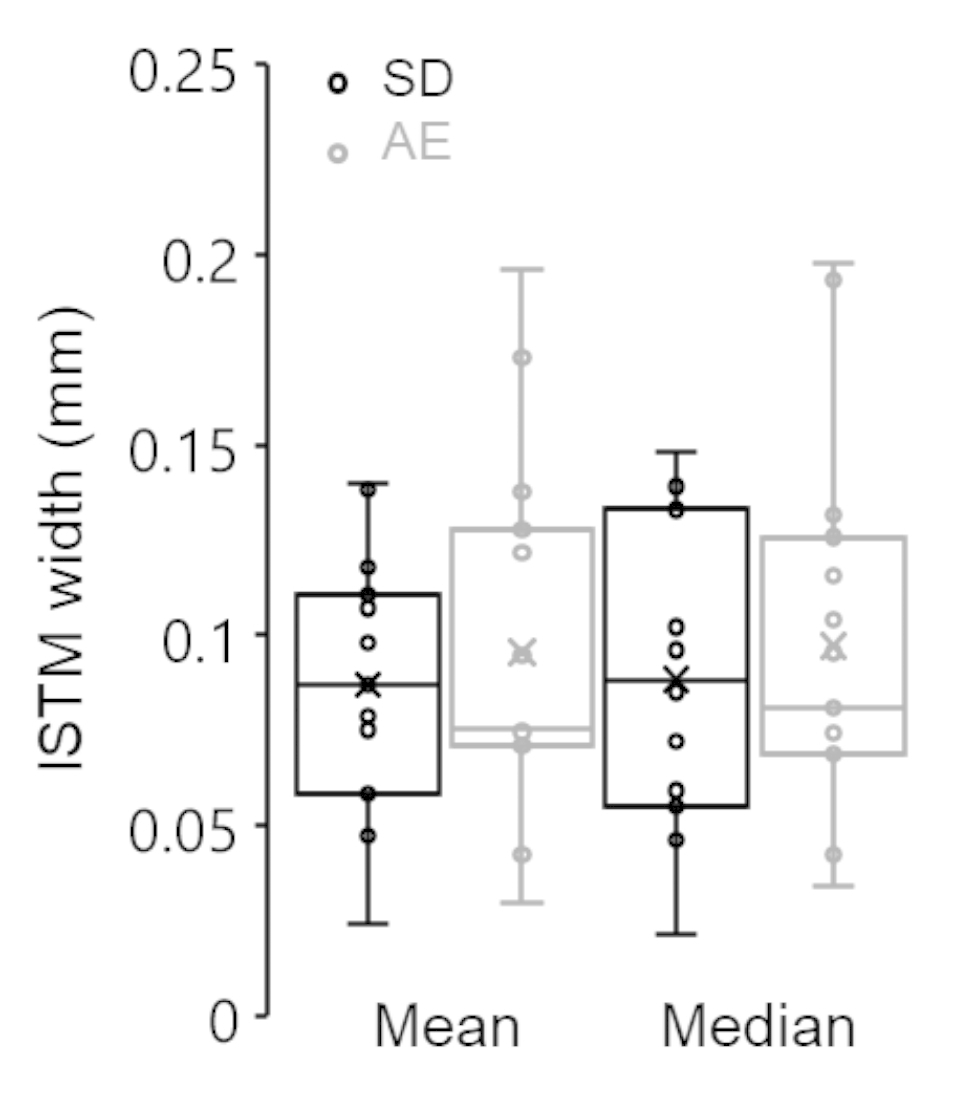
*Box-Whisker plots of ISTM width from ORO and/or filipin stained sections (n = 32; 16 SD, 16 ApoE*^*−/−*^*[AE]). No significant differences in ISTM width found betwee*n *rat strains (p = 0.349)*

Intra-rater reliability of grading ORO-stained sections was good to excellent (ICC = 0.839, 95% CI = 0.727–0.906) and that of filipin-stained sections was excellent (ICC = 0.981, 95% CI = 0.965–0.990). After grading of ORO staining, 6 sections were omitted for poor confidence scores, hence 26 sections were included in the statistical analysis. Confidence scores did not differ between groups (Mean ± s.d. ORO: SD 3.2 ± 0.6 and *ApoE*^*−/−*^ 3.2 ± 0.8; Filipin: SD 3.6 ± 0.7 and *ApoE*^*−/−*^ 3.3 ± 0.8). Both rat strains demonstrated positive ORO staining in the ISTM (Fig. 2) and fat pad (Suppl. Fig. 1) but rarely between fibres (Fig. 2). By contrast, both *ApoE*^*−/−*^ and SD rats demonstrated positive and diffuse filipin staining amongst fibres (Fig. 2) but rarely in the ISTM (Fig. 2). On occasion, intracellular staining and well-defined lipid deposits were observed in both groups (Suppl. Fig. 1). No significant differences were found in any staining scores between groups (*p* = 0.196–0.801). Frequency histograms of grading scores are presented in Fig. 3. ISTM width did not differ significantly between rat strains (Fig. 4, p = 0.349).

### Tissue biomechanics

Both Achilles tendons of 32 rats (16 SD, 16 *ApoE*^*−/−*^) were prepared for biomechanical testing. Nine tendons were damaged during sample dissection and preparation, leaving 55 samples prepared for biomechanical testing. Of the samples tested, 2 tests could not be analysed and 15 samples demonstrated evidence of fibre crossover at test termination and therefore excluded from further analysis [9]. Thus, cyclic load and force relaxation data were collated on 38 samples (18 SD, 20 *ApoE*^*−/−*^). Of these, 10 samples were excluded from the ramp to failure test analyses for reaching the load cell’s software-implemented overload stop, therefore 14 SD and 16 *ApoE*^*−/−*^ samples available for investigation of quasi-static failure properties.

**Fig. 5:**
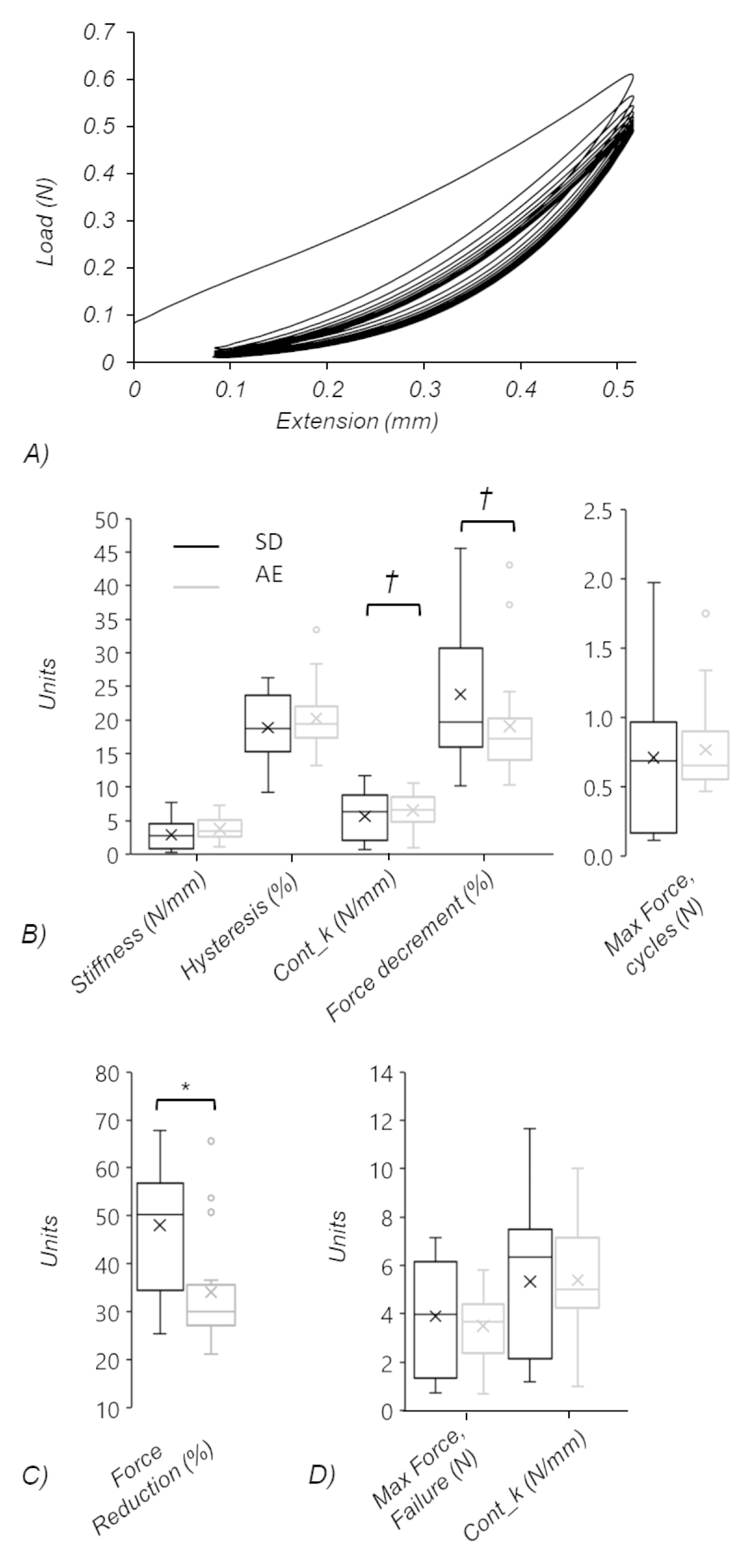
(A) Example load-extension curve from cyclic test. (B-D) Box-Whisker plots showing the data quartiles, mean (x) and outliers (o) for variables calculated from (B) cyclic tests (note two y-axes), (C) force relaxation tests, and (D) ramp-to-failure tests

Differences were found in biomechanical properties between rat groups; % force reduction measured from the force relaxation test (Fig. 5) was significantly greater in SD rats (*p* = 0.005). From the cyclic tests (Fig. 5), continuous stiffness trended higher, and cyclic force decrement (%) trended lower, in *ApoE*^*−/−*^*rats* (*p* = 0.051 and 0.099, respectively). We found no differences between strains in any other properties. Post-hoc calculations using final sample sizes found properties other than stiffness and force relaxation were underpowered for detecting between group differences, hence there is an inflated possibility of type II error and our results should be interpreted with caution.

To help provide confidence that sub-tendon differences did not impact our results, we confirmed there were no differences in the mechanical and viscoelastic properties of SD and *ApoE*^*−/−*^ tail fascicles (due to the nature of the ISTM shear test, we were unable examine the properties of Achilles sub-tendons) using the same loading parameters as described for the ISTM testing in this study (data not reported).

### External strain analysis

An analysis of sub-tendon strains during shear testing was carried out during the cyclic testing of 16 randomly chosen samples (8 SD, 8 *ApoE*^*−/−*^). Grip-to-grip sample starting length (at 0.1 N pre-load) was 8.3 ± 1.2 and 7.9 ± 1.5 mm for SD and *ApoE*^*−/−*^, respectively, and the cyclically applied absolute displacement for all samples was 0.5 mm. Sub-tendon marker coordinates were used to estimate local strains in the GM (GM1-GM2), SOL (SOL1-SOL2) in response to the gross applied strain, and to detect the extent of sliding between the sub-tendons. Marker displacements within the sub-tendons were 0.05 ± 0.06 mm, which translated to sub-tendon strains of 4.5 ± 3.6%. With sub-tendon strains only ~ 1/10th of the applied displacement, we conclude that the majority of the extension occurred through ISTM shearing, but also note that the ISTM is mechanically robust.

## Discussion

The first aim of this study was to examine whether a high blood cholesterol environment would translate to greater intratendinous lipid content in the Achilles tendon. We used an *ApoE*^*−/−*^ rat model to mimic familial hypercholesterolemia and found these rats, when fed a regular chow diet, had double the total blood cholesterol of our SD rats at euthanasia. However this was only half of that expected [33] and therefore our model demonstrated only a mild hypercholesterolemia phenotype.

We expected to find differences between the *ApoE*^*−/−*^ and SD rats localised to the ISTM region, with a higher lipid content in the proteoglycan-rich ISTM of high cholesterol animals. However, we found evidence of both esterified and unesterified lipids in the Achilles tendons of both rat groups, and the amounts and locations of the lipids did not differ notably between *ApoE*^*−/−*^ and SD rats. ORO staining varied considerably between rats and demonstrated that intra-tendinous esterified lipids were often - but not always - found in the ISTM. ORO staining in the extracellular space between collagen fibres was rarely found presently but has been previously reported in hypercholesterolemic tendons [40, 41]. Filipin staining demonstrated that unesterified lipids were diffuse and present between collagen fibres. Previous studies noting this same staining pattern also indicate that staining is more intense in high-fat diet fed hypercholesterolemia models [40, 42]. Unesterified cholesterol-rich lipid particles are reported to constitute the initial lipid deposition in atherosclerotic lesion development and so are of particular interest for examining the link between high cholesterol and tendon pathology. As the pathophysiological mechanisms underpinning tendon xanthoma development are hypothesized to be similar to atherosclerosis [4], we expected these cholesterols to be visualized in areas containing blood vessels [41], rather than in a diffuse nature. We did note small concentrated unesterified lipid deposits between fibres, in the ISTM (Fig. 2) and intracellularly in a minority of animals (Suppl. Fig. 1) and no such deposits were found in the vasculature-rich peritendon. It is interesting to note that esterified (ORO) and unesterified (filipin) cholesterol staining were almost mutually exclusive [40] as both cholesterols are equally found in xanthomas [43]. It would be of future interest to examine the association between blood cholesterol level and tendon cholesterol extent using an appropriate high cholesterol model.

Histological analysis typically relies on sample imaging from only a few locations within the tissue and so provides a limited assessment of overall tendon cholesterol content and distribution. Moreover, fresh-frozen tissue sections are often of a lower quality than formalin fixed paraffin embedded tissue sections, and may be affected by sectioning artifacts, particularly ISTM width measurements. However, given the number of sections that were available for ISTM width measures, and the consistency with which they were treated, we assume that any artifact in measurement was consistent between groups.

Our second hypothesis was that the presence of tendon cholesterol would alter ISTM mechanical properties. Here, it is of note that despite a lack of observable differences in ISTM cholesterol between groups, we found significant differences in ISTM behaviour. Specifically, *ApoE*^*−/−*^ rats demonstrated a trend towards increased stiffness as well as reduced cyclic force decrement and force-relaxation properties. With consistency in sample sizes, applied displacements and measured forces, data indicates that differences did not arise from test conditions, but alterations in viscoelastic tissue properties. All tendons demonstrate a degree of viscoelasticity, providing mechanisms to modulate tissue strain response to different loading conditions. With viscoelasticity generally considered to be related to proteoglycan or water content, it seems reasonable to hypothesize that the reduction in viscoelasticity and trend in increased stiffness in *ApoE*^*−/−*^ rats is associated with ISTM changes.

The ISTM displays a distinctive protein composition, with proteoglycans (notably lubricin) to facilitate sliding and elastin to facilitate recoil [9, 13, 44, 45]. These molecules have previously been investigated for interactions with lipids. Elastin - the core molecule of elastic fibres that impart extensibility and elastic recoil ability to the extracellular matrix - has also been implicated in atherosclerotic plaque progression [46]. Although elastin’s mechanical and viscoelastic properties are hydration-dependent (Wang et al. 2018), elastin is a hydrophobic protein and an attractive location for depositing hydrophobic ligands, such as cholesterol [47]. The binding of elastin to lipids (as occurs in atherosclerosis) has been shown to alter elastin chain flexibility [48] as well as impede the interaction between water and elastin [49] - two mechanisms that might be expected to impact ISTM mechanics. Like elastin, GAGs also have a high affinity for lipids. We might have hypothesized *ApoE*^*−/−*^ rats to have a greater GAG content than control rats had these animals demonstrated a higher tendon lipid content, as both LDL and oxidized LDL (oxLDL) can alter GAG chain synthesis to enhance its lipoprotein binding properties [50–52]. Surprisingly, the removal of GAGs in bovine tendons led to more stress relaxation and greater reductions in failure stress, indicating the relationship between GAG content and viscoelasticity is not direct [20]. If increased lipids enhance GAG content in *ApoE*^*−/−*^ rats, this may contribute to reduced force relaxation. However, we did not quantify ISTM composition in the current study and so these discussion points are purely speculative. Perhaps there are subtle differences in the composition or distribution of matrix proteins and complexes between our rat groups that were not distinguishable using the basic histological methods employed here. Techniques such as laser capture microscopy could be used to explore associations between ISTM composition and high cholesterol environments in future.

Studies investigating the link between hypercholesterolemia and tendon health report changes in biomechanical properties that are associated with tendon pathology and injury prevalence [2, 53]. In the current study, few biomechanical differences were found in ISTM properties between groups despite hypothesising that sub-tendon sliding would be impacted with tendon pathology [10, 54]. However, post-hoc analyses using our final samples found many variables were underpowered for detecting differences between groups and our findings should be interpreted with caution.

While subtle changes in biomechanical properties may suffice to increase injury prevalence with mild hypercholesterolemia, our young *ApoE*^*−/−*^ rat model fed a regular diet was not ideal for examining tendon xanthoma development. A better model for testing our hypotheses may have been achieved by providing a high fat diet to enhance the lipid profiles of our *ApoE*^*−/−*^ rats [55, 56], although increased mortality may have been an issue [33, 56]. Increased evidence of atherosclerosis and xanthoma development in other species with ageing [41, 57] suggests a longer exposure to high blood cholesterol levels [53, 58] increases the likelihood of developing tendon lipid deposits. Such models would better reflect familial hypercholesterolemia in humans [41, 59] and would be necessary for examining our hypotheses with clarity.

## Conclusion

Hypercholesterolemia is associated with tendon pathology, but the reasons underpinning this relationship are not well understood. We hypothesized that elevated blood cholesterol would be associated with an increased presence of tendon cholesterol, which in turn would impact on tendon biomechanical properties, but did not find sufficient evidence to support our hypothesis. Our *ApoE*^*−/−*^ rats only demonstrated a mild hypercholesterolemia phenotype, which did not translate to a visibly increased Achilles tendon lipid content. However, it is notable that even with this mild phenotype, force relaxation was significantly lower in *ApoE*^*−/−*^ than SD rats, highlighting the possibility that even slightly elevated cholesterol may result in subtle changes to tendon biomechanical properties and hence injury risk. Nonetheless, we conclude that the young, *ApoE*^*−/−*^ rat is not a suitable model to study the impact of tendon xanthomas on Achilles tendon function.

## Electronic supplementary material

Below is the link to the electronic supplementary material.

Supplementary Material 1

## Data Availability

Images and datasets used and/or analysed during the current study are available from the corresponding author on reasonable request.
