# Supplementary figures and images for: Mild hypercholesterolemia impacts achilles sub-tendon mechanical properties in young rats
Source: BMC Musculoskelet Disord. 2023 Apr 12;24:282. doi: 10.1186/s12891-023-06375-0 (PMC10091839; doi:10.1186/s12891-023-06375-0)

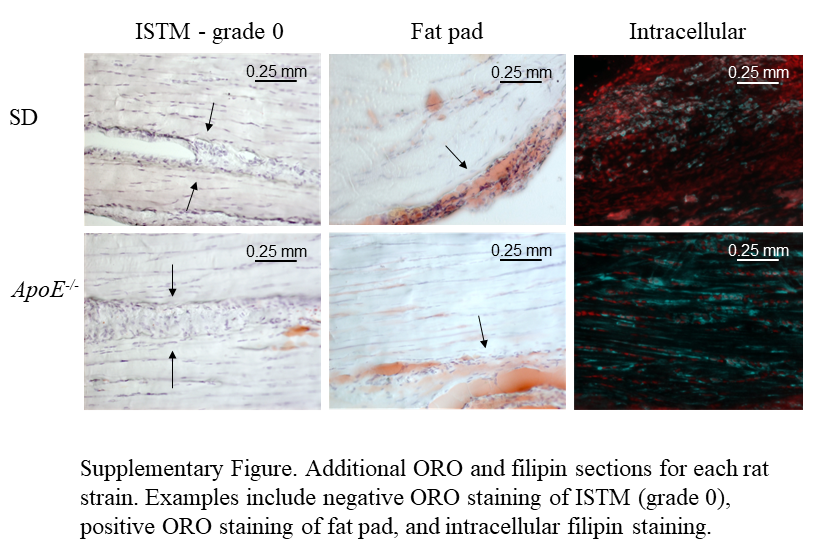

Supplement: Supplementary file 1 — Supplementary Material 1 [file 12891_2023_6375_MOESM1_ESM.png]
